# Viscosity Investigations on the Binary Systems of (1 ChCl:2 Ethylene Glycol) DES and Methanol or Ethanol

**DOI:** 10.3390/molecules26185513

**Published:** 2021-09-10

**Authors:** Reza Haghbakhsh, Ana Rita C. Duarte, Sona Raeissi

**Affiliations:** 1 School of Chemical and Petroleum Engineering, Shiraz University, Mollasadra Ave., Shiraz 71348-51154, Iran; r.haghbakhsh@fct.unl.pt; 2 LAQV, REQUIMTE, Departamento de Química da Faculdade de Ciências e Tecnologia, Universidade Nova de Lisboa, 2829-516 Caparica, Portugal; ard08968@fct.unl.pt

**Keywords:** Deep Eutectic Solvent, eutectic mixture, green solvent, alcohol, alkanol, physical property, excess property, viscosity deviation, thermodynamic modeling

## Abstract

In this study, the viscosity behavior of two mixtures of Ethaline (1 ChCl:2 ethylene glycol) with either methanol or ethanol were investigated over the temperature range of 283.15–333.15 K at atmospheric pressure. The measured viscosities of neat Ethaline, methanol, and ethanol showed reliable agreement with the corresponding reported literature values. The mixture viscosities were modeled by an Arrhenius-like model to determine the behavior of viscosity with respect to temperature. The data were also modeled by the four well-known mixture viscosity models of Grunberg–Nissan, Jouyban–Acree, McAllister, and Preferential Solvation. All of the model results were reliable, with the Jouyban–Acree and Preferential Solvation models showing the most accurate agreement with the experimental measurements. The Jones–Dole viscosity model was also investigated for the measured viscosities, and by analyzing the results of this model, strong interactions among Ethaline and the alcohol molecules were proposed for both systems. As a final analysis, viscosity deviations of the investigated systems were calculated to study the deviations of the viscosity behaviors with respect to ideal behavior. Both systems showed negative viscosity deviations at all of the investigated temperatures, with the negative values tending towards zero, and hence more ideal behavior, with increasing temperatures. Moreover, in order to correlate the calculated viscosity deviations, the Redlich–Kister model was successfully used for both systems and at each investigated temperature.

## 1. Introduction

The concept of Deep Eutectic Solvents (DESs) was introduced by Abbott et al. [1] in 2003 as a novel generation of green solvents with somewhat similar properties to Ionic Liquids (ILs). Early investigations on DESs [2,3,4,5,6,7,8,9,10,11] revealed a number of favorable properties as green solvents, such as biodegradability, sustainability, low toxicity, negligible vapor pressure, and good solvation power. These properties are the ones that resemble those of most ILs; however, in addition, DESs are generally cheaper and easier to prepare than ILs. Yet, not all DESs are as ideal as one would wish for, as some have been shown to have volatility and toxicity in the literature [9,10,11]. Despite many similarities in properties, DESs are a different chemical category from ILs. A DES is actually an associating mixture of at least two components consisting of a hydrogen bond acceptor (HBA) and a hydrogen bond donor (HBD), while an IL is a pure component involving ionic interactions between its constituent cations and anions. In this manner, they are theoretically quite different green solvents, having some similarities in their general behavior [12].

The hydrogen bond interactions, which are dominant in DES solutions, are very complicated [13]. This calls for investigation, especially regarding recently-introduced DESs.

The complexity of hydrogen bond interactions is amplified when the DES is mixed with yet another associating component, such as an alcohol which itself accommodates hydrogen bonds. How the hydrogen bond networks of the DES and alcohol are changed, and whether new hydrogen bonds are established between the DES and alcohol, are the main questions arising for such solutions. One of the methods to provide initial answers to these questions is to investigate and compare the physical properties of the DES and alcohol in their pure state and also in solution. Density and viscosity are the most important, yet easy to measure physical properties, which can provide clues to answer these questions.

Furthermore, viscosity in itself is a fundamental physical property whose values must be available for any fluid before modeling, simulation, and design can be carried out. In this respect, the generally high values of viscosities for most DESs are a major drawback for their widespread use [14]. The addition of water or other conventional solvents, such as alcohols, to DESs is a method to reduce their viscosities, especially very highly viscous DESs [15]. Therefore, it is necessary to expand our knowledge on viscosity changes with concentration in DES solutions. Furthermore, the investigation of the viscosity behavior of DESs with conventional solvents, such as alcohols, provides clues on the hydrogen bond changes. Up to now, only a few limited studies [16,17,18,19] have been devoted to the viscosity behavior of DESs mixtures with alcohols.

In 2016, Sas et al. were the first group who investigated the viscosity behavior of DES mixtures with alcohols. They chose the choline chloride: levulinic acid (1:2) DES in mixture with either of the normal alcohols of ethanol, 1-propanol, 1-butanol, or 1-pentanol at the three temperatures of 298.15, 308.15, and 318.15 K. They reported negative viscosity deviations for all of the investigated systems with respect to ideal mixture viscosities [17]. Gajardo-Parra et al., in 2019, investigated the three choline chloride-based DESs of ChCl: levulinic acid, ChCl:ethylene glycol, and ChCl:phenol at the same molar ratio of 1:2 in mixture with 1-butanol within the temperature range of 293.15–333.15 K. They also reported negative viscosity deviations in the mixtures with respect to the pure DES and 1-butanol [18]. In the present year, Wang et al. investigated the two well-known choline chloride based DESs of ChCl:ethylene glycol and ChCl:glycerol at the molar ratio of 1:2 in mixtures with methanol within the temperature range of 288.15–323.15 K. According to the measured data, they also calculated excess molar volumes, viscosity deviations, and excess molar Gibbs energies of activation for the investigated systems. They reported negative values for excess volumes, as well as negative viscosity deviations at all of the investigated temperatures and compositions [19]. Recently, Jangir et al. investigated the viscosity behavior of the ChCl:lactic acid (1:2) DES in mixture with ethanol or ethylene glycol. They presented negative viscosity deviations in these mixtures with respect to the neat viscosities for both of the investigated systems [16].

The limited number of studies on the viscosities of DES mixtures with alcohols, in comparison to the large number of introduced DESs, and the importance of alcohols in the chemical industries show the significant gap in the literature on this topic. Therefore, in this study, the viscosity behavior of Ethaline (1 ChCl:2 ethylene glycol), as one of the less viscous and most commonly used DESs, is determined experimentally in mixtures with the two common alcohols of methanol and ethanol over the temperature range of 283.15–333.15 K and at atmospheric pressure. Then, the four well-established models of Grunberg–Nissan [20], Jouyban–Acree [21], McAllister [22] and Preferential Solvation [23,24] were developed according to the experimental measurements. These models can then be considered as practical engineering tools to estimate the viscosities of the investigated mixtures at any composition desired.

## 2. Experimental Procedures

### 2.1. Chemicals

The detailed information of the compounds used in this study including vendor, purity, and purification methods are presented in Table 1. Choline chloride, because of its high moisture adsorbing characteristic, was dried in a vacuum oven at a temperature of 60 °C for 24 h. The other compounds, because of their initial high purity, were used in the experiments without further purification.

### 2.2. Deep Eutectic Solvent Preparation

Ethaline, which is the combination of one mole of choline chloride and two moles of ethylene glycol, was prepared by precise weighing of the dried choline chloride and ethylene glycol using a digital balance by Shimadzu UW1020H, with an uncertainty of 0.001 g. After mixing the weighed amounts of dried choline chloride and ethylene glycol into a sealed vial, the mixture was kept in a shaker-incubator for 24 h at a temperature of 60 °C. Then, the mixture was placed in a vacuum oven for 24 h at the temperature of 60 °C to remove the possibly absorbed moisture during preparation. In this way, Ethaline, which is a homogeneous transparent liquid, was prepared. In order to determine the water content of the prepared Ethaline, tests were carried out using a Metrohm 787 KF Titrino Karl-Fischer titrator. The amount of water in Ethaline was recorded as 0.00421 in mass fraction, which is acceptable for a DES.

### 2.3. Mixture Samples Preparation

Nine pseudo-binary mixture samples over the whole composition range of Ethaline + methanol and Ethaline + ethanol were prepared at step sizes of about 0.1 in molar composition. The required amounts of Ethaline and methanol/ethanol were weighed with an uncertainty of 0.001 g by the digital balance mentioned above, and collected in closed-top vials.

### 2.4. Viscosity Measurements

Viscosities of the prepared samples of Ethaline + methanol and Ethaline + ethanol were measured by an Anton Paar SVM^TM^ 3000 viscometer. Before the measurements, the viscometer was calibrated by the Anton Paar set of standard oils. The relative uncertainty of viscosity measurements was 0.35%. The viscosities of the Ethaline + methanol and Ethaline + ethanol samples were measured at temperatures ranging from 283.15 to 323.15 and 293.15 to 333.15 K, respectively. Five temperature steps, with intervals of 10 K, were considered for each temperature range. In addition to the mixture samples, the viscosities of pure Ethaline, methanol, and ethanol were measured at the corresponding temperatures. The uncertainty of temperature measurement was 0.02 K. All of the measurements were carried out at the atmospheric pressure of 100 kPa, with an uncertainty of 5 kPa.

## 3. Theory

The modeling of the investigated Ethaline + methanol/ethanol systems could be divided into two parts. First, viscosity estimations of the mixtures at different compositions and temperatures were investigated, and then, the viscosity deviations of the mixtures were treated.

### 3.1. Mixture Viscosity Models

#### 3.1.1. Arrhenius-Type Model

The Arrhenius-type model is the simplest and most commonly used model for correlating the viscosities of liquids, whether in the pure state or as mixtures. Equation (1) presents this model [25].

(1) $$\eta =\eta_{0}\exp(\frac{-E_{a}}{RT})$$

This model does not directly consider the effect of concentration of the mixture on the liquid viscosity, and only has a functionality of temperature. For each composition, the parameters of *η*_0_ and *E_a_*, which are the reference viscosity and activation energy parameter, respectively, are optimized to the experimental viscosity data. R is the universal gas constant. Although this equation is limited to a specific composition, it can be used as a very simple viscosity model for thermodynamic derivations.

To optimize the parameters of *η*_0_ and *E_a_*, Equation (2) is considered as the objective function,

(2) $$Ob.Fun=\sum_{i}^{n}\frac{\left|\eta_{mix,i}^{exp.}-\eta_{mix,i}^{cal.}\right|}{\eta_{mix,i}^{exp.}}$$

where $\eta_{mix,i}^{exp.}$ and $\eta_{mix,i}^{cal.}$ are the experimental and calculated mixture viscosities by the model, respectively, and n is the number of investigated data pints.

#### 3.1.2. Preferential Solvation Theory

The preferential solvation theory proposes the definition of mutual complex molecules or associated molecules in a mixture of molecules *i* and *j*. The mutual complex molecule, or better called associated molecules, in DESs (*ij* or *ii* or *jj*) is formed by the HBA (*S_i_*) and HBD (*S_j_*) because of the prevailing strong hydrogen bonds. Equations (3)–(5) formulate the solvation equations of some possible interactions [24],

(3) $${\left(S_{i}^{L}\right)}_{m}+mS_{j}\overset{g_{j/i}}{↔}{\left(S_{j}^{L}\right)}_{m}+mS_{i}$$

(4) (SiL)m+m2Sj↔gij/i(SijL)m+m2Si

(5) (SiL)m+m2Sjj↔gjj/i(SjjL)m+m2Si

where subscript *L* symbolizes local molecules, $g_{j/i}$, $g_{ij/i}$ and $g_{jj/i}$ are the preferential solvation parameters, and m is the average number of molecules in a mutual complex molecule. The general expression of this theory follows:

(6) $$\eta_{mix}=\sum_{i=1}^{N}x_{i}^{L}\eta_{i}^{0}+\sum_{i=1}^{N}\sum_{j=1}^{N}x_{ij}^{L}\eta_{ij}\;$$

In this equation, $\eta_{mix}$, $\eta_{i}^{0}$ and $\eta_{ij}$ are the mixture, the pure compound, and the mutual associated dynamic viscosities, respectively. By expanding the general form of Equation (6), different interactions in the mixture are considered. However, according to the nature and type of molecules involved, some interactions are dominant and the less important interactions may be canceled from the general form. For a binary liquid mixture of a molecular solvent (*i* = 1) and a DES (*j* = 2), the self-association interactions of the DES (22) are dominant in this theory, thus Equation (6) is simplified to Equation (7) [24].

(7) $$\eta_{mix}=x_{1}^{L}\eta_{1}^{0}+x_{2}^{L}\eta_{2}^{0}+x_{22}^{L}\eta_{22}$$

In this equation, $x_{1}^{L}$, $x_{2}^{L}$ and $x_{22}^{L}$ are the local compositions, which are calculated as follows [24].

(8) x1L=(1-x2)m(1−x2)m+g2/1(x2)m+gij/1[(1−x2)x2]m2

(9) x2L=g2/1(x2)m(1−x2)m+g2/1(x2)m+gij/1[(1−x2)x2]m2

(10) xijL=gij/1[(1−x2)x2]m2(1−x2)m+g2/1(x2)m+gij/1[(1−x2)x2]m2

By inserting Equations (8)–(10) into Equation (7), the final viscosity model of the preferential solvation theory for the binary mixture of a molecular solvent and a DES is derived.

(11) ηmix=η1+g2/1(η2−η1)(x2)m+gij/1(ηij−η1)[(1−x2)x2]m2(1−x2)m+g2/1(x2)m+gij/1[(1−x2)x2]m2

The parameter m is considered as 2.5 [24], and the three preferential solvation parameters of $g_{2/1}$, $g_{ij/1}$ and $\eta_{ij}$ are fit to the experimental data at each temperature based on Equation (2) as the objective function. In order to avoid optimization for each temperature set individually, it is recommended to use instead, simple temperature-dependent functions for the three preferential solvation parameters as:

(12) $$g_{2/1}=g_{2/1}^{p}+g_{2/1}^{q}T$$

(13) $$g_{ij/1}=g_{ij/1}^{p}+g_{ij/1}^{q}T$$

(14) $$\eta_{ij}=\eta_{ij}^{p}+\eta_{ij}^{q}T$$

#### 3.1.3. Grunberg–Nissan Viscosity Model

A correlation for the viscosities of binary mixtures was proposed by Grunberg–Nissan as [20]:

(15) $$\ln(\eta_{mix})=x_{1}\ln(\eta_{1})+x_{2}\ln(\eta_{2})+x_{1}x_{2}G_{ij}$$

where $\eta_{mix}$ is the mixture dynamic viscosity, $\eta_{1}$ and $\eta_{2}$ are the pure dynamic viscosities of components 1 and 2, respectively, *x*_1_ and *x*_2_ are the molar compositions of components 1 and 2, respectively, and $G_{ij}$ is a temperature-dependent binary interaction energy parameter that should be optimized for each temperature using the experimental data, based on Equation (2) as the objective function. However, in order to avoid over-fitting at each temperature, it is recommended to incorporate a simple temperature-dependent expression for $G_{ij}$ as follows.

(16) $$G_{ij}=G_{1}+G_{2}T$$

#### 3.1.4. McAllister Viscosity Model

The McAllister model, with a four-body form, considers the activation energy of molecular motion [22,24]:

(17) $$\begin{array}{l} \ln(\eta_{mix})=x_{1}^{4}\ln(\eta_{1})+4x_{1}^{3}x_{2}\ln(\eta_{1112})+6x_{1}^{2}x_{2}^{2}\ln(\eta_{1122})+4x_{1}x_{2}^{3}\ln(\eta_{2221})+x_{2}^{4}\ln(\eta_{2})-ln(x_{1}+x_{2}\frac{Mw_{2}}{Mw_{1}})\\ +4x_{1}^{3}x_{2}\ln(\frac{\left(3+\frac{Mw_{2}}{Mw_{1}}\right)}{4})+6x_{1}^{2}x_{2}^{2}\ln(\frac{\left(1+\frac{Mw_{2}}{Mw_{1}}\right)}{2})+4x_{1}x_{2}^{3}\ln(\frac{\left(1+\frac{3Mw_{2}}{Mw_{1}}\right)}{4})+x_{2}^{4}\ln(\frac{Mw_{2}}{Mw_{1}}) \end{array}$$

where $\eta_{mix}$ is the mixture dynamic viscosity, $\eta_{1}$ and $\eta_{2}$ are the pure dynamic viscosities of the molecular solvent and the DES, respectively, $Mw_{1}$ and $Mw_{2}$ are the molecular weights of the molecular solvent and the DES, respectively, and $\eta_{1112}$, $\eta_{1122}$, and $\eta_{2221}$ are the interaction parameters of the model. These interaction parameters are adjusted based on the experimental data for each temperature set, utilizing Equation (2) as the objective function for optimization. It is better to propose one simple temperature-dependent function for each.

(18) $$\eta_{1112}=K_{1}+K_{2}T$$

(19) $$\eta_{1122}=K_{3}+K_{4}T$$

(20) $$\eta_{2221}=K_{5}+K_{6}T$$

#### 3.1.5. Jouyban–Acree Viscosity Model

The Jouyban–Acree viscosity model is a simple viscosity model for mixtures. For binary liquid mixtures, it takes the following form [21]:

(21) $$\ln(\eta_{mix})=x_{1}\ln(\eta_{1})+x_{2}\ln(\eta_{2})+A_{0}\left(\frac{x_{1}x_{2}}{T}\right)+A_{1}\left(\frac{x_{1}x_{2}(x_{1}-x_{2})}{T}\right)+A_{2}\left(\frac{x_{1}x_{2}{\left(x_{1}-x_{2}\right)}^{2}}{T}\right)$$

where $\eta_{mix}$ is the mixture dynamic viscosity, $\eta_{1}$ and $\eta_{2}$ are the pure dynamic viscosities of the molecular solvent and the DES, respectively, and *A*_0_, *A*_1_ and *A*_2_ are the interactions parameters of the model which are fit based on the experimental data, with Equation (2) as the objective function.

#### 3.1.6. Jones–Dole Viscosity Model

The Jones–Dole viscosity model is an empirical model that presents the relative viscosity of a binary mixture as a function of the DES molar concentration [26,27]:

(22) $$\frac{\eta_{mix}}{\eta_{0}}=1+A\sqrt{C}+B\times C$$

where $\eta_{mix}$ and $\eta_{0}$ are the mixture and the pure molecular solvent dynamic viscosities, respectively, *C* is the concentration of DES in the mixture in molar basis, and *A* and *B* are the Falkenhagen coefficient and viscosity B-coefficient, respectively [28].

The Falkenhagen coefficient denotes the solute–solute interactions, and is calculated theoretically. However, it is usually considered to be negligible for nonelectrolyte solutions [27,28]. Then, Equation (22) is simplified for a pseudo-binary mixture of DES + molecular solvent as [27]:

(23) $$\frac{\eta }{\eta_{0}}=1+B\times C$$

According to this equation, the viscosity *B*-coefficient is calculated as the slope of relative viscosity ($\frac{\eta }{\eta_{0}}$) with respect to the DES molar concentration (*C*). By analyzing the values of this coefficient at different temperatures, some information about the DES–molecular solvent interactions is obtained [29].

### 3.2. Viscosity Deviation Model

In addition to having the tools to estimate the mixture viscosity of the investigated systems at any temperature and composition, as given above, it is also valuable to look into the behavior of mixture viscosity for insight on the potential interactions within the mixture. To do this, one can investigate the viscosity deviation of a mixture, $\Delta \eta_{mix}$, defined as the difference between a mixture property and its ideal solution value:

(24) $$\Delta \eta_{mix}=(\eta_{mix})-[x_{1}(\eta_{1})+x_{2}(\eta_{2})]$$

This property is an indicator to investigate how the mixture viscosity changes with respect to the molar-based arithmetic average of its individual constituent viscosities. In this equation, $\eta_{1}$ and $\eta_{2}$ are the pure constituent viscosities of the molecular solvent and DES, respectively, at the same temperature as the desired mixture viscosity, and *x*_1_ and *x*_2_ are the molar compositions of the molecular solvent and the DES, respectively. In order to model the viscosity deviation, the Redlich–Kister expansion, being one of the best-established models for excess properties, is used here. For a binary mixture, the Redlich–Kister model is [30]:

(25) $$ln(\Delta \eta_{mix})=x_{1}x_{2}\sum_{j=0}^{k}D_{i}{\left(x_{1}-x_{2}\right)}^{j}$$

where the *D_i_*’s are the Redlich–Kister coefficients, and their number is dependent on the desired value of k. At any temperature, these coefficients are optimized to the calculated values of viscosity deviations from the experimental data, using Equation (26) as the objective function. Depending on the number of experimental data available at any temperature, this series can be expanded to the desired number of sentences by choosing the parameter *k*. In this study, the value of *k* was considered as three, in order to produce four sentences for the Redlich–Kister series.

(26) $$Ob.Fun^{\Delta }=\sum_{i}^{n}\frac{\left|\Delta \eta_{mix,i}^{exp.}-\Delta \eta_{mix,i}^{cal.}\right|}{\Delta \eta_{mix,i}^{exp.}}$$

In this equation, $\Delta \eta_{mix,i}^{exp.}$ and $\Delta \eta_{mix,i}^{cal.}$ are the experimental viscosity deviation and calculated viscosity deviation by the Redlich–Kister model, respectively, and *n* is the number of investigated data.

## 4. Results and Discussion

**Figure 1:**
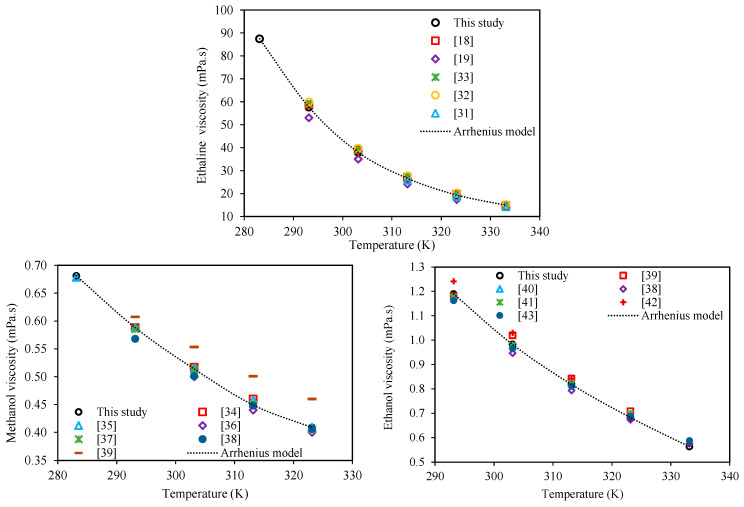
Comparison of the behaviors of the measured viscosities with respect to temperature at atmospheric pressure for Ethaline (1 ChCl:2 ethylene glycol), methanol, and ethanol in this study and literature values (dotted lines are the Arrhenius models, Equation (1)).

As the first step for experimental measurements of the investigated mixture viscosities, in addition to viscometer calibration with the standard oils, the viscometer measurements were further validated on the pure compounds, for which viscosity data is available in the literature. Table 2 compares the viscosities for Ethaline, methanol, and ethanol measured in this study to the corresponding literature values within the temperature range of 283.15–333.15 K at atmospheric pressure. Besides the quantitative values given in this table, Figure 1 shows a qualitative comparison of the measured and literature viscosities with temperature. According to Table 2 and Figure 1, the measured viscosity values for Ethaline, methanol, and ethanol are in very good agreement with the literature values and their trends. This agreement is particularly noteworthy for Ethaline, because usually, the prepared DESs in different laboratories have various water contents which can affect the values of their viscosities.

**Figure 2:**
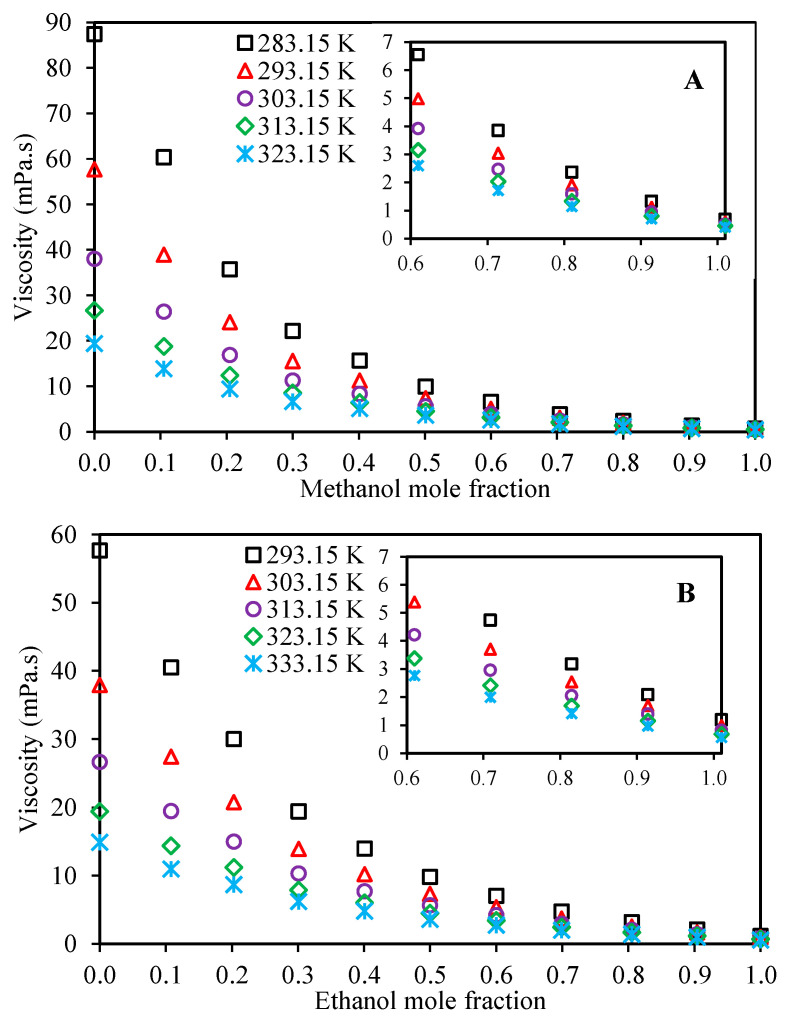
Viscosity behavior of the mixtures with respect to alcohol concentration for the investigated systems at a pressure of 100 kPa ((**A**): Ethaline (1 ChCl:2 ethylene glycol) + methanol, (**B**): Ethaline (1 ChCl:2 ethylene glycol) + ethanol).

Table 3 presents the measured experimental values for the viscosities of Ethaline + methanol and Ethaline + ethanol at the investigated temperatures and atmospheric pressure over the whole composition range. Figure 2 illustrates the mixture viscosity behavior with respect to composition. As expected, by increasing the concentrations of methanol or ethanol, the viscosity of the mixture decreases. These depressions in viscosity follow exponential-like trends for both the Ethaline + methanol/ethanol systems. According to this figure, it is obvious that the temperature has significant effects on the viscosities, especially in the Ethaline-rich mixtures, and in general, increasing alcohol concentrations can dramatically change the viscosities of the mixture. This can be used to our advantage in various applications to reduce the viscosities of Ethaline for greater applicability in the industries.

In addition to viscosity, in order to have a comprehensive overview, the reported values of densities for the investigated mixtures of Ethaline + methanol/ethanol are presented in Table S1 of the Supplementary data.

In order to calculate the viscosities of the investigated mixtures at various temperatures and compositions, several well-known models were introduced in the Theory section. These models can be divided into two categories. The first category is the Arrhenius-like viscosity model (Equation (1)), which does not consider any concentration changes; therefore, this model must be fit for each concentration separately. Table 4 presents the optimized values for the parameters of the Arrhenius-type model at each investigated composition for both Ethaline + methanol/ethanol systems. Additionally, the values of Average Absolute Relative Deviation percent (AARD%), which show the accuracy of the model with respect to experimental data, were calculated according to Equation (27) and presented in this table.

(27) $$AARD\%=\frac{100}{N}\sum_{i}^{n}\frac{\left|\eta_{mix,i}^{exp.}-\eta_{mix,i}^{cal.}\right|}{\eta_{mix,i}^{exp.}}$$

**Figure 3:**
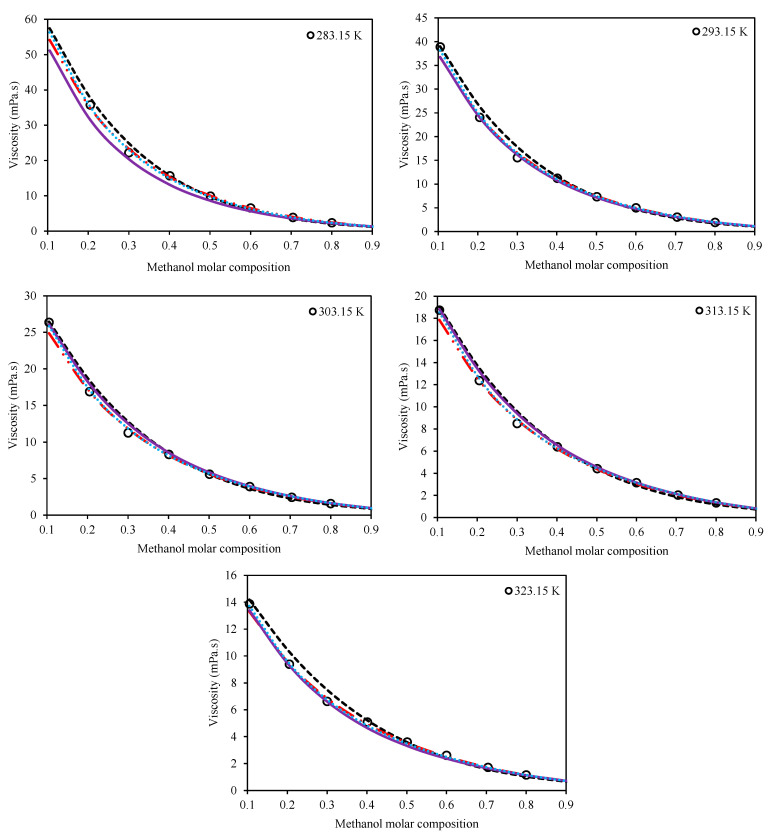
Comparison of the behavior of the Grunberg–Nissan [20], Jouyban–Acree [21], McAllister [22], and Preferential Solvation [23,24] models with respect to the experimental trend for the system of Ethaline (1 ChCl:2 ethylene glycol) + methanol at various temperatures and at a pressure of 100 kPa. (Grunberg–Nissan (---), Jouyban–Acree (―••), McAllister (―), Preferential Solvation (•••), Experimental data (o)).

**Figure 4:**
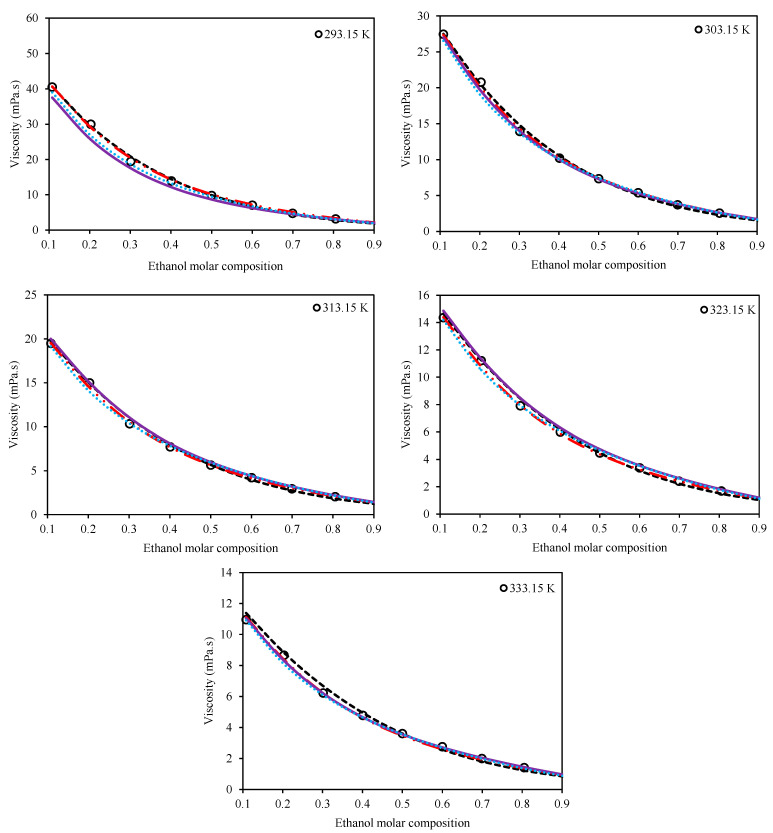
Comparison of the behavior of the Grunberg–Nissan [20], Jouyban–Acree [21], McAllister [22], and Preferential Solvation [23,24] models with respect to the experimental trend for the system of Ethaline (1 ChCl:2 ethylene glycol) + ethanol at various temperatures and at a pressure of 100 kPa. (Grunberg–Nissan (**---**), Jouyban–Acree (**―••**), McAllister (**―**), Preferential Solvation (**•••**), Experimental data (**o**)).

The second category consists of the models which consider both the temperature and composition of the mixture for viscosity estimations. The Preferential Solvation (Equation (11)), Grunberg–Nissan (Equation (15)), Jouyban–Acree (Equation (21)), and McAllister (Equation (17)) viscosity models belong to this category. Table 5 presents the optimized values of the adjustable parameters for these four models for both Ethaline + methanol/ ethanol. In order to have a comparison among the models, the values of AARD% are also presented for each system in this table, according to Equation (27). Figure 3 and Figure 4 present a qualitative comparison of the trends of the investigated models for Ethaline + methanol and Ethaline + ethanol, respectively. As expected, the Preferential Solvation model, because of its richer theoretical background, has better results and trends for both investigated systems. The Jouyban–Acree model, although a simple model, achieves surprising results of quite compatible mathematical trends with respect to the actual experimental trends. The McAllister model, which has a complicated mathematical expression containing many adjustable parameters, shows less reliable results than the Preferential Solvation model with the same number of adjustable parameters (both models have six fitted parameters), and even the Jouyban–Acree with a smaller number of parameters.

Finally, as expected, the Grunberg–Nissan model, because of its very simple mathematical expression and the least number of adjustable parameters, showed the least reliable results. However, it should be considered that all of the investigated models, according to Table 5 and Figure 3 and Figure 4, generally produced acceptable results and provide good viscosity estimations for both of the investigated systems.

As explained, the relative viscosity of the mixture (ratio of mixture viscosity to the viscosity of pure alcohol) is represented by the well-known Jones–Dole model (Equation (23)). Table 6 presents the calculated values of the viscosity B-coefficients of this model by the least squares method for both pseudo-binary systems of Ethaline + methanol/ethanol at various temperatures and compositions. The most important values of the viscosity B-coefficients are their values at infinite dilution of alcohol in the mixture. According to this table, the viscosity B-coefficients have positive values at all of the investigated temperatures for both systems, which indicates the presence of strong interactions among the DES and alcohol molecules in the mixture. Moreover, it is clear from the table that the values of viscosity B-coefficients decrease with increasing temperatures for both systems, which is expected. By increasing the temperature, the interactions among the DES and alcohol molecules decrease, leading to decreased viscosity B-coefficients. Since this coefficient is actually a kind of indicator of the strength of molecular interactions in the mixture, it can successfully predict the effect of the hydrogen bond strength with respect to temperature in the systems. Moreover, if we compare the values of the viscosity B-coefficients of Ethaline + methanol to those of Ethaline + ethanol at the same temperature, it is seen that at each temperature, this value is higher for Ethaline + methanol than for Ethaline + ethanol, which probably shows stronger hydrogen bond interactions among Ethaline and methanol molecules than between Ethaline and ethanol molecules.

**Figure 5:**
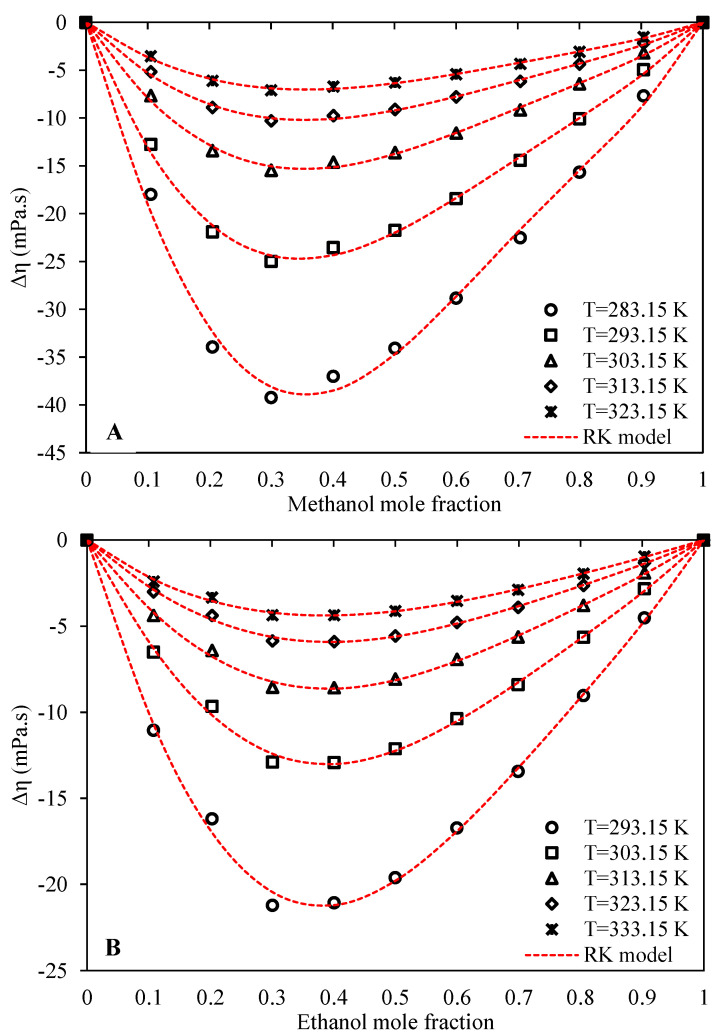
Behavior of viscosity deviation from ideality with respect to alcohol concentration at the pressure of 100 kPa (A: Ethaline (1 ChCl:2 ethylene glycol) + methanol, B: Ethaline (1 ChCl:2 ethylene glycol) + ethanol).

For clues on molecular interactions in a mixture, one important method is to study the excess properties. The values of viscosity deviations from ideality of both the systems of Ethaline + methanol/ethanol were calculated based on the measured experimental viscosities using Equation (24), and presented in Table S2 of Supplementary data. Figure 5 provides a graphical overview of the viscosity deviations with respect to composition for both systems. It is seen that the calculated viscosity deviations are negative for both systems at all of the investigated temperatures and compositions. This indicates that the viscosity of the mixture is less than the molar-based arithmetic average of the viscosities of neat Ethaline and either of the alcohols. In other words, both investigated systems are strongly non-ideal regarding viscosity, with negative deviations from the ideal state. Viscosity is generally a very challenging property to understand and model, and there is still no comprehensive theory for describing viscosity mechanisms in the liquid phase [44]. There are many parameters which affect the liquid viscosity, and this complexity is even more highlighted for mixtures. However, molecular interactions and interstitial accommodations are perhaps the two most important mechanisms responsible for viscosity deviations from the ideal state in the liquid phase. The unknowns in this field are so great that one can even observe a striking contrast among various literature studies. While some studies claim that stronger interactions within the mixture as compared to the pure components lead to negative values of viscosity deviation [16,19,44,45], others claim that stronger mixture interactions lead to positive viscosity deviations [15,46,47,48]. In their interesting paper, Friend and Hargreaves [48] claimed that increasing numbers of association interactions and their strength will increase the viscosities of liquids, while Viswanath et al. [44], in their book, claimed that the increase of any interactions in the liquid phase, such as polar or association interactions, results in decreased viscosities. Probably, the reason for this inconsistency is that academics may actually be neglecting other important phenomena that may increase or decrease, or even appear or disappear upon changes in association bonds resulting from the process of mixing. For example, interstitial accommodation is just one of these phenomena, whereby the number of voids and their size can increase or decrease by changes in the association numbers and their strength.

The interstitial accommodation can either increase or decrease the viscosity according to the nature of components. For example, if the small molecules completely fill the void spaces of the larger molecules, being placed within the structure of larger molecules (for example between the branches of a branched molecule), the molecular size discrepancy of the mixture is decreased, possibly leading to more facile flow, and hence, decreasing viscosity. However, if the smaller molecules do not make a nearly perfect fit with the void spaces of the large molecules, with parts of the smaller molecule sticking out, this creates some “branches” protruding out of the large molecules, resulting in resistance to flow and increased viscosities.

According to our measurements and results for both systems, the values of viscosity deviations from ideality plummeted to even further negativity by decreasing the temperature, which shows that the rate of viscosity increase by decreasing temperature is higher for pure Ethaline and alcohols with respect to the mixture. If the temperature is lowered, both systems tend to more non-ideal states, likely with stronger hydrogen bond interactions between the unlike molecules. A similar behavior was observed by other researchers for different DES mixtures [16,19].

A comparison of excess volume between the two systems at the same temperature shows that the values are slightly more negative for Ethaline + methanol than for Ethaline + ethanol, hinting at the possibly stronger interactions and more non-ideal behavior for the Ethaline + methanol system. This is consistent with the proposed discussion on the viscosity B-coefficients of the Jones–Dole model, where we suggested stronger interactions for the Ethaline + methanol system. However, methanol, because it is of smaller size than ethanol, may possibly result in better-packed interstitial accommodations, leading to greater viscosity reduction for its mixtures. Both systems, however, show quite similar trends of viscosity deviation with respect to concentration, with the minimum viscosity deviations in the Ethaline-rich region, occurring at an alcohol molar composition of about 0.3 to 0.4.

The Redlich–Kister model (Equation (25)) was used to correlate the viscosity deviations with respect to concentration at each investigated temperature. Table 7 presents the optimized values of the Redlich–Kister parameters for both systems.

In order to have a quantitative index for the accuracies of the fitted Redlich–Kister models, the corresponding values of AARD%s are also presented in Table 7. The graphical behavior and trends of the model are illustrated in Figure 5 using dashed curves. The fitted Redlich–Kister models have good accuracies and reliable trends with respect to the experimental viscosity deviation values, as indicated by both the AARD% values and the correct graphical trends.

## 5. Conclusions

Among the important gaps of literature on the physical properties of DESs is the scarce number of studies on viscosity investigations of DES mixtures with alcohols, especially by taking into account the importance of alcohols in the chemical industries. This gap is even more highlighted when considering the insignificant number of studies as compared to the large number of DESs already introduced. This study investigated the viscosity behavior of the two systems of Ethaline (1 ChCl:2 ethylene glycol) + methanol or ethanol. Before the mixture viscosity measurements, validations with literature values were carried out at all the desired temperatures using pure Ethaline, as well as pure methanol and ethanol. Nine mixtures of different compositions were prepared for each system over the entire concentration range and their viscosities were measured, in addition to those of neat Ethaline, methanol, and ethanol. The data covered the temperature range of 283.15–333.15 K at atmospheric pressure. The behavior of viscosity with respect to temperature was modeled by an Arrhenius-like model at each investigated composition. Furthermore, the behavior of viscosity as functions of both composition and temperature was modeled by the four models of Grunberg–Nissan [20], Jouyban–Acree [21], McAllister [22], and Preferential Solvation [23,24]. The results were reliable, with AARD% values of 7.08, 2.31, 4.81, and 2.61% for the Ethaline + methanol system and 5.66, 2.41, 4.38, and 3.95% for the Ethaline + ethanol system, according to the Grunberg–Nissan, Jouyban–Acree, McAllister, and Preferential Solvation models, respectively. The surprisingly excellent accuracy of the Jouyban–Acree model, by incorporating only three adjustable parameters for each system, was the significant result of the model comparison. Furthermore, for more insight, the Jones–Dole viscosity model was applied to both systems and the important parameter of viscosity B-coefficient was calculated at each investigated temperature. The positive values of the viscosity B-coefficients suggested strong interactions among the DES and alcohol molecules in both mixtures. By comparing the viscosity B-coefficients of the two systems with one another, it was suggested that the Ethaline + methanol system probably has the stronger hydrogen bond interactions than does Ethaline + ethanol. Finally, the important parameter of viscosity deviation from ideality was calculated for the investigated systems at each temperature to show their viscosity deviations from the ideal state. Negative viscosity deviations were obtained for both systems at all of the investigated temperatures. These negative values of viscosity deviations tended towards zero upon increasing the temperature for both systems. The reasons for the negative sign, and also for the reduction of negativity upon increasing temperatures, are possibly the interactions of two of the most important mechanisms involved, i.e., the association interactions and the interstitial accommodations within the mixture. Each mechanism can move the mixture viscosity behavior towards or away from the ideal state. However, most probably, the stronger interactions between Ethaline and the methanol/ethanol molecules in the mixture with respect to the neat interactions of Ethaline, methanol, and ethanol in their pure states have the most significant effect on the negative values of viscosity deviations. The Redlich–Kister model was further applied successfully to correlate the viscosity deviations of both systems with respect to composition at all of the investigated temperatures.

## Figures and Tables

**Table 1:** Purities, vendors, and purification methods of the chemicals used in this study.

Chemical Name	CAS Number	IUPAC Name	Source	Initial Mass Fraction Purity	Purification Method
Choline chloride	67-48-1	2-hydroxyethyl (trimethyl) azanium chloride	Acros Organics	99%	Dried 24 h in oven vacuum
Ethylene glycol	107-21-1	Ethane-1,2-diol	Merck	99.5%	No further purification
Methanol	67-56-1	Methanol	Merck	99.9%	No further purification
Ethanol	64-17-5	Ethanol	Merck	99.9%	No further purification

**Table 2:** Comparison of experimental values of viscosities for Ethaline, methanol, and ethanol in this study with literature values at atmospheric pressure ^a,b,c^.

Reference	*T* (K)					
	283.15	293.15	303.15	313.15	323.15	333.15
Ethaline (1 ChCl:2 Ethylene Glycol)	*μ* (mPa.s)					
This study	87.449	57.664	37.968	26.649	19.403	14.913
Brennecke et al. [18]	-	58.81	38.9	27.04	19.74	14.69
Harifi-Mood and Buchner [31]	-	-	-	25.8	18.8	14.2
Gajardo-Parra et al. [32]	-	60	39.78	27.77	20.3	15.31
Wang et al. [19]	-	53.07	35.06	24.22	17.41	-
Crespo et al. [33]	-	59.144	39.101	27.439	19.697	14.76
**Methanol**	***μ* (mPa.s)**					
This study	0.681	0.588	0.514	0.449	0.409	-
Qian et al. [34]	-	0.588	0.517	0.460	-	-
Fan et al. [35]	0.6781	0.5884	0.5154	0.4576	0.4093	-
Gomez Marigliano and Solimo [36]	-	-	0.5	0.44	0.4	-
McAtee and Heitz [37]	-	0.5853	0.5122	0.4508	0.4051	-
Gong et al. [38]	-	0.568	0.501	0.448	0.406	-
Kurnia and Mutalib [39]	-	0.6075	0.5535	0.501	0.4601	-
**Ethanol**	***μ* (mPa.s)**					
This study	-	1.189	0.982	0.818	0.682	0.562
Kurnia and Mutalib [39]	-	1.1802	1.0191	0.8424	0.708	-
Lu et al. [40]	-	1.177	0.978	0.822	0.698	-
Gong et al. [38]	-	1.141	0.946	0.793	0.673	0.576
Zhang et al. [41]	-	1.177	0.978	0.822	0.698	-
Xu et al. [42]	-	1.241	1.029	0.842	-	-
Mokhtarani et al. [43]	-	1.1617	0.9645	0.81	0.6868	0.5872

^a^ Standard uncertainties u are u(T) = 0.02 K, u(p) = 5 kPa and the relative uncertainty for dynamic viscosity is ur(η) = 0.35%. ^b^ The composition of the prepared Ethaline and its standard uncertainty is (0.333 ± 0.001 Choline chloride + 0.667 ± 0.002 ethylene glycol) in mole fraction. ^c^ Molecular weight of Ethaline is 87.92 g·mol^−1^.

**Table 3:** Experimental viscosities of Ethaline + methanol/ethanol at the investigated temperatures and at a pressure of 100 kPa ^a,b,c^.

*T* (K)	Viscosity (mPa.s)										
	*x*_1_ Ethaline (1 ChCl:2 Ethylene Glycol) + *x*_2_ Methanol										
*x*_2_	0.000	0.105	0.205	0.300	0.401	0.501	0.600	0.704	0.800	0.904	1.000
283.15	87.449	60.352	35.719	22.186	15.643	9.906	6.545	3.846	2.358	1.327	0.681
293.15	57.664	38.905	24.059	15.539	11.225	7.323	4.986	3.039	1.917	1.112	0.588
303.15	37.968	26.368	16.887	11.264	8.336	5.604	3.915	2.459	1.588	0.944	0.514
313.15	26.649	18.744	12.376	8.497	6.407	4.426	3.157	2.033	1.338	0.810	0.449
323.15	19.403	13.864	9.387	6.617	5.073	3.585	2.600	1.711	1.142	0.701	0.409
	***x*** **_1_** ** Ethaline (1 ChCl:2 ethylene glycol) + *x*_2_ ethanol**										
*x*_2_	0.000	0.108	0.203	0.301	0.401	0.500	0.600	0.699	0.805	0.904	1.000
293.15	57.664	40.517	30.000	19.439	13.936	9.809	7.050	4.739	3.170	2.090	1.189
303.15	37.968	27.461	20.789	13.929	10.208	7.347	5.390	3.708	2.535	1.701	0.982
313.15	26.649	19.484	14.994	10.328	7.712	5.654	4.221	2.960	2.053	1.395	0.818
323.15	19.403	14.375	11.222	7.912	6.000	4.469	3.384	2.409	1.690	1.159	0.682
333.15	14.913	10.961	8.661	6.226	4.787	3.612	2.766	1.994	1.411	0.973	0.562

^a^ Standard uncertainties u are u(T) = 0.02 K, u(p) = 5 kPa and the relative uncertainty for dynamic viscosity is ur(η) = 0.35%. ^b^ The composition of the prepared Ethaline and its standard uncertainty is (0.333 ± 0.001 Choline chloride + 0.667 ± 0.002 ethylene glycol) in mole fraction. ^c^ Molecular weight of Ethaline is 87.92 g·mol^−1^.

**Table 4:** The optimized values of the Arrhenius-like viscosity model (Equation (1)), and AARD% for the investigated mixtures of Ethaline + methanol/ethanol at each investigated concentration.

Parameters	Viscosity (mPa.s)										
	*x*_1_ Ethaline (1 ChCl:2 Ethylene Glycol) + *x*_2_ Methanol										
*x*_2_	0.000	0.105	0.205	0.300	0.401	0.501	0.600	0.704	0.800	0.904	1.000
η_0_ (mPa.s)	4.137 × 10^−−4^	2.877 × 10^−4^	5.516 × 10^−4^	1.002 × 10^−3^	1.441 × 10^−3^	2.280 × 10^−3^	3.351 × 10^−3^	5.145 × 10^−3^	6.501 × 10^−3^	7.641 × 10^−3^	1.009 × 10^−2^
E_a_ (j·mol^−1^)	−2.886 × 10^4^	−2.883 × 10^4^	−2.607 × 10^4^	−2.354 × 10^4^	−2.186 × 10^4^	−1.971 × 10^4^	−1.783 × 10^4^	−1.557 × 10^4^	−1.387 × 10^4^	-1.214 × 10^4^	−9.910 × 10^3^
AARD%	2.09	1.92	1.50	1.29	1.17	1.08	0.85	0.60	0.35	0.03	0.56
	***x*_1_** **Ethaline (1 ChCl:2 Ethylene Glycol) + *x*_2_ Ethanol**										
*x*_2_	0.000	0.108	0.203	0.301	0.401	0.500	0.600	0.699	0.805	0.904	1.000
η_0_ (mPa.s)	4.137 × 10^−4^	5.671 × 10^−4^	7.546 × 10^−4^	1.233 × 10^−3^	1.633 × 10^−3^	2.121 × 10^−3^	2.668 × 10^−3^	3.313 × 10^−3^	3.656 × 10^−3^	3.619 × 10^−3^	2.680 × 10^−3^
E_a_ (j·mol^−1^)	−2.886 × 10^4^	−2.723 × 10^4^	−2.580 × 10^4^	−2.355 × 10^4^	−2.205 × 10^4^	−2.056 × 10^4^	−1.920 × 10^4^	−1.770 × 10^4^	−1.649 × 10^4^	−1.550 × 10^4^	−1.487 × 10^4^
AARD%	2.09	1.55	1.35	1.05	0.92	0.74	0.59	0.40	0.17	0.12	0.94

**Table 5:** The optimized values of adjustable parameters and AARD% values for the Grunberg–Nissan [20], Jouyban–Acree [21], McAllister [22], and Preferential Solvation [23,24] models for viscosity estimation of Ethaline (1 ChCl:2 ethylene glycol) + methanol/ethanol systems at a pressure of 100 kPa.

**System**	**Viscosity Models**						**AARD%**
	**Grunberg–Nissan Parameters**						
	**G_1_**			**G_2_**			
Ethaline + methanol	0.7853			0.0006			7.08
Ethaline + ethanol	−0.5244			0.0042			5.66
	**Jouyban−Acree Parameters**						
	**A_0_**		**A_1_**		**A_2_**		
Ethaline + methanol	300.2		334.3		90.9		2.39
Ethaline + ethanol	238.7		267.0		294.7		2.41
	**McAllister Parameters**						
	**K_1_**	**K_2_**	**K_3_**	**K_4_**	**K_5_**	**K_6_**	
Ethaline + methanol	18.85	−0.0518	44.17	−0.1281	134.79	−0.3911	4.81
Ethaline + ethanol	26.11	−0.07	34.72	−0.0961	125.16	−0.3519	4.38
	**Preferential Solvation Parameters**						
	$g_{2/1}^{p}$	$g_{2/1}^{q}$	$g_{ij/1}^{p}$	$g_{ij/1}^{q}$	$\eta_{ij}^{p}$	$\eta_{ij}^{q}$	
Ethaline + methanol	−0.1240	0.0011	0.9166	0.0025	29.2126	−0.0807	2.61
Ethaline + ethanol	−0.1120	0.0010	0.4148	0.0037	48.1058	−0.1328	3.95

**Table 6:** The calculated values of the viscosity B-coefficients of the Jones–Dole model for Ethaline + methanol/ethanol mixtures at various temperatures and at a pressure of 100 kPa.

*T* (K)	*B* (cm^3^/mol)										
	*x*_1_ Ethaline (1 ChCl:2 Ethylene Glycol) + *x*_2_ Methanol										
*x*_2_	0.000	0.105	0.205	0.300	0.401	0.501	0.600	0.704	0.800	0.904	1.000
283.15	9.956	7.226	4.520	2.978	2.275	1.577	1.167	0.789	0.576	0.425	-
293.15	7.627	5.403	3.527	2.412	1.885	1.342	1.021	0.714	0.533	0.404	-
303.15	5.757	4.193	2.831	1.996	1.595	1.168	0.909	0.653	0.497	0.382	-
313.15	4.630	3.412	2.372	1.719	1.398	1.051	0.834	0.613	0.474	0.370	-
323.15	3.708	2.773	1.973	1.466	1.211	0.928	0.747	0.558	0.434	0.333	-
	***x*_1_** **Ethaline (1 ChCl:2 Ethylene Glycol) + *x*_2_ Ethanol**										
*x*_2_	0.000	0.108	0.203	0.301	0.401	0.500	0.600	0.699	0.805	0.904	1.000
293.15	3.394	2.561	2.050	1.442	1.146	0.906	0.751	0.589	0.493	0.439	-
303.15	2.702	2.097	1.714	1.244	1.009	0.814	0.687	0.550	0.470	0.427	-
313.15	2.292	1.796	1.490	1.111	0.917	0.753	0.646	0.527	0.458	0.422	-
323.15	2.024	1.605	1.351	1.031	0.863	0.720	0.627	0.521	0.460	0.434	-
333.15	1.940	1.525	1.300	1.012	0.861	0.729	0.645	0.547	0.494	0.486	-

**Table 7:** The optimized values of the Redlich–Kister coefficients for the Ethaline + methanol/ethanol mixtures at various temperatures and at a pressure of 100 kPa.

*T* (K)	RK Coefficients				AARD%
	*D*_0_	*D*_1_	*D*_2_	*D*_3_	
	Ethaline (1 ChCl:2 Ethylene Glycol) + Methanol				
283.15	–138.90	104.700	–25.080	–53.040	4.55
293.15	–88.00	63.130	–24.280	–16.740	3.17
303.15	–55.05	38.020	–13.390	–12.280	3.37
313.15	–36.78	24.560	−9.314	–7.159	3.18
323.15	–25.46	16.470	–6.583	–4.796	3.14
	**Ethaline (1 ChCl:2 Ethylene Glycol) + Ethanol**				
293.15	–79.14	45.440	–5.825	–14.750	2.26
303.15	–48.93	26.440	–1.325	–10.430	2.40
313.15	–32.53	16.940	–1.238	–5.771	2.38
323.15	–22.42	11.270	–0.792	–3.596	2.14
333.15	–16.53	8.249	–1.792	–0.646	2.24

## Data Availability

All relevant data are included within the manuscript.

## Supplementary Materials

Table S1: Reported values of experimental densities of pseudo-binary mixtures of (1-x) Ethaline + x methanol/ethanol at various temperatures and at a pressure of 100 ± 5 kPa [References R1, R2]. Table S2: Calculated values of viscosity deviations from ideality for Ethaline + methanol/ethanol systems at various temperatures at a pressure of 100 kPa.

## Sample Availability

Samples of the compounds are not available from the authors.
