# Supplementary material for: Viscosity Investigations on the Binary Systems of (1 ChCl:2 Ethylene Glycol) DES and Methanol or Ethanol
Source: Molecules. 2021 Sep 10;26(18):5513. doi: 10.3390/molecules26185513 (PMC8465307; doi:10.3390/molecules26185513)
Supplement: Supplementary file 1 [file molecules-26-05513-s001.zip › molecules-1336267-supplementary.pdf]

molecules

Supplementary Information

Viscosity Investigations on the Binary Systems of (1 ChCl:2 Ethylene Glycol) DES and Methanol or Ethanol

Reza Haghbakhsh [1,2], Ana Rita C. Duarte [2] and Sona Raeissi [1,*]

1 School of Chemical and Petroleum Engineering, Shiraz University, Mollasadra Ave., Shiraz 71348-51154, Iran; r.haghbakhsh@fct.unl.pt
2 LAQV, REQUIMTE, Departamento de Química da Faculdade de Ciências e Tecnologia, Universidade Nova de Lisboa, 2829-516 Caparica, Portugal; ard08968@fct.unl.pt
* Correspondence: raeissi@shirazu.ac.ir

Table S1. Reported values of experimental densities of pseudo-binary mixtures of (1-x) Ethaline + x methanol/ethanol at various temperatures and at a pressure of 100±5 kPa [References R1, R2].

Reference	x	Temperature (K)				
		**283.15**	**293.15**	**303.15**	**313.15**	**323.15**
		ρ ($g.cm^{-3}$)				
		(1-x) **Ethaline +** x **methanol**				
**[R1]**	0.000	1.1252	1.1192	1.1133	1.1077	1.1021
[R1]	0.105	1.1118	1.1059	1.1001	1.0944	1.0888
[R1]	0.205	1.0950	1.0888	1.0828	1.0769	1.0711
[R1]	0.300	1.0780	1.0717	1.0656	1.0595	1.0534
[R1]	0.401	1.0564	1.0500	1.0437	1.0376	1.0313
[R1]	0.501	1.0318	1.0250	1.0185	1.0120	1.0056
[R1]	0.600	1.0035	0.9968	0.9898	0.9829	0.9762
[R1]	0.704	0.9663	0.9588	0.9516	0.9444	0.9373
[R1]	0.800	0.9237	0.9168	0.9092	0.9015	0.8937
[R1]	0.904	0.8692	0.8607	0.8523	0.8440	0.8355
[R1]	1.000	0.8007	0.7911	0.7817	0.7721	0.7626
		**293.15**	**303.15**	**313.15**	**323.15**	**333.15**
		(1- x) **Ethaline +** x. **ethanol**				
[R2]	0.000	1.1192	1.1133	1.1077	1.1021	1.0965
[R2]	0.101	1.0984	1.0926	1.0868	1.0811	1.0753
[R2]	0.182	1.0806	1.0741	1.0682	1.0623	1.0566
[R2]	0.308	1.0487	1.0424	1.0363	1.0302	1.0242
[R2]	0.401	1.0231	1.0165	1.0102	1.0039	0.9979
[R2]	0.501	0.9932	0.9865	0.9799	0.9734	0.9668
[R2]	0.598	0.9622	0.9552	0.9483	0.9415	0.9347
[R2]	0.700	0.9250	0.9176	0.9104	0.9032	0.8961
[R2]	0.798	0.8848	0.8772	0.8696	0.8621	0.8543
[R2]	0.894	0.8433	0.8352	0.8271	0.8190	0.8106
[R2]	1.000	0.7915	0.7828	0.7741	0.7652	0.7559

Table S2. Calculated values of viscosity deviations from ideality for Ethaline + methanol/ethanol systems at various temperatures at a pressure of 100 kPa.

T **(K)**	**Viscosity deviation (mPa.s)**										
	**x_1 Ethaline (1 ChCl : 2 ethylene glycol) + x_2 methanol**										
x_2	**0.000**	**0.105**	**0.205**	**0.300**	**0.401**	0.501	0.600	0.704	0.800	0.904	1.000
283.15	0.000	−17.986	−33.943	−39.233	−37.012	−34.072	−28.843	−22.519	−15.677	−7.684	0.000
293.15	0.000	−12.766	−21.904	−25.002	−23.551	−21.746	−18.433	−14.444	−10.086	−4.955	0.000
303.15	0.000	−7.667	−13.403	−15.468	−14.612	−13.599	−11.580	−9.141	−6.416	−3.166	0.000
313.15	0.000	−5.154	−8.902	−10.292	−9.736	−9.096	−7.773	−6.171	−4.352	−2.155	0.000
323.15	0.000	−3.545	−6.123	−7.088	−6.713	−6.302	−5.407	−4.320	−3.065	−1.531	0.000
	**x_1 Ethaline (1 ChCl : 2 ethylene glycol) + x_2 ethanol**										
x_2	0.000	0.108	0.203	0.301	0.401	0.500	0.600	0.699	0.805	0.904	1.000
293.15	0.000	−11.048	−16.200	−21.226	−21.081	−19.617	−16.729	−13.449	−9.031	−4.520	0.000
303.15	0.000	−6.513	−9.671	−12.906	−12.929	−12.128	−10.387	−8.407	−5.659	−2.832	0.000
313.15	0.000	−4.375	−6.411	−8.546	−8.579	−8.080	−6.929	−5.634	−3.802	−1.903	0.000
323.15	0.000	−3.006	−4.381	−5.856	−5.896	−5.573	−4.787	−3.908	−2.643	−1.320	0.000
333.15	0.000	−2.402	−3.339	−4.368	−4.372	−4.125	−3.536	−2.888	−1.950	−0.967	0.000

References:

[R1] Haghbakhsh, R.; Raeissi, S. Experimental investigation on the volumetric properties of mixtures of the deep eutectic solvent of Ethaline and methanol in the temperature range of 283.15 to 323.15 K. *J. Chem. Thermodyn*. **2020,** *147*, 106124.

[R2] Haghbakhsh, R.; Raeissi, S. A study of nonideal mixtures of ethanol and the 1 choline chloride + 2 ethylene glycol deep eutectic solvent for their volumetric behavior. *J. Chem. Thermodyn*. **2020,** *150*, 106219.
